# Hybrid Prediction Method for ECG Signals Based on VMD, PSR, and RBF Neural Network

**DOI:** 10.1155/2021/6624298

**Published:** 2021-03-15

**Authors:** Fuying Huang, Tuanfa Qin, Limei Wang, Haibin Wan

**Affiliations:** ^1^School of Electronic and Information Engineering, South China University of Technology, Guangzhou 510641, China; ^2^School of Computer, Electronics and Information, Guangxi University, Nanning 530004, China; ^3^Guangxi Meteorological Information Center, Nanning 530022, China

## Abstract

To explore a method to predict ECG signals in body area networks (BANs), we propose a hybrid prediction method for ECG signals in this paper. The proposed method combines variational mode decomposition (VMD), phase space reconstruction (PSR), and a radial basis function (RBF) neural network to predict an ECG signal. To reduce the nonstationarity and randomness of the ECG signal, we use VMD to decompose the ECG signal into several intrinsic mode functions (IMFs) with finite bandwidth, which is helpful to improve the prediction accuracy. The input parameters of the RBF neural network affect the prediction accuracy and computational burden. We employ PSR to optimize input parameters of the RBF neural network. To evaluate the prediction performance of the proposed method, we carry out many simulation experiments on ECG data from the MIT-BIH Arrhythmia Database. The experimental results show that the root mean square error (RMSE) and mean absolute error (MAE) of the proposed method are of 10^−3^ magnitude, while the RMSE and MAE of some competitive prediction methods are of 10^−2^ magnitude. Compared with other several prediction methods, our method obviously improves the prediction accuracy of ECG signals.

## 1. Introduction

ECG signals are very important for doctors to diagnose diverse kinds of heart diseases. It is of significance to predict ECG signals accurately. Accurate prediction of ECG signals can help doctors know the patient's condition in advance, while it can also reduce the energy consumption of sensors in body area networks (BANs). In BANs, some sensors are placed under the skin or inside the body, and their batteries are inconvenient to replace. How to reduce the energy consumption and prolong the lifetime of such sensors is a challenge. If a prediction model is established in both a sensor node and the sink node, when the prediction error exceeds the specified threshold value, the sensor node will send the measured data; otherwise, it will not send the measured data, and the sink node will use the predicted data as the measured data [1]. In other words, prediction can reduce the volume of transmitted data [2], thus reducing the energy consumption of the sensor. In BANs, there are many physiological signals, such as the ECG, body temperature, and blood pressure. If these physiological signals can be accurately predicted from the past and current data, the amount of data transmission and the energy consumption of the sensor will be greatly reduced.

There are many methods of predicting physiological signals. Among them, the artificial neural network (ANN) has become a widely used prediction method because of its nonlinearity, self-adaption, and fast calculation speed. As a type of ANN, a radial basis function (RBF) neural network has the advantages of fast training speed and ability to not easily fall into the local optimum; thus, RBF neural networks have attracted increasingly more attention in time series prediction [3–6]. The key problem of designing a RBF neural network is to determine the network structure parameters, including the center of the hidden layer, base width, and weight of the output layer. In recent years, several algorithms have been proposed to solve the problem of parameter optimization [7–10]. To solve global optimization problems, an enhanced MSIQDE (improved quantum-inspired differential evolution) algorithm based on mixing multiple strategies was proposed in [7]. This algorithm has better optimization ability than the DE (differential evolution), QDE (quantum-inspired differential evolution), QGA (quantum genetic algorithm), and MSIQDE algorithms. In [8], a novel improved DE algorithm with the wavelet basis function was proposed. This algorithm shows better optimization ability than other several DE algorithms and effectively solved a real-world airport gate assignment problem. To optimize the parameters of photovoltaic models and enhance the conversion efficiency of solar energy, Song et al. [9] proposed a new multipopulation parallel coevolutionary DE algorithm that showed higher accuracy and reliability than other several methods in extracting parameters of solar modules.

When using a RBF neural network to predict time series signals, it is not enough to only optimize its internal structure parameters. One must also optimize the input parameters of its input layer. The input parameters of a RBF neural network include input dimension and delay time. Different input parameters produce different prediction results. It is difficult to determine the optimal input parameters. Phase space reconstruction (PSR) [11] is one of the effective methods used to solve the input parameter optimization problem of RBF neural networks. PSR reconstructs the multidimensional phase space structure of the original system through a one-dimensional time series signal. The embedding dimension and delay time of PSR are taken as the input dimension and delay time of the neural network, respectively. Several researchers have used PSR to optimize the input parameters of neural networks and obtained good prediction results [12–14].

This paper focuses on the prediction of ECG signals. In BANs, the ECG signal has the largest amount of data among all physiological signals. The sensor will consume a significant amount energy to transmit vast quantities of raw ECG data. To save the energy of the sensor, it is necessary to reduce the transmission of ECG data without affecting the normal use of ECG signals. An ECG signal is a nonlinear nonstationary time series signal and has an inherent random feature. Although an ECG signal is considered a periodic signal, its period does not keep a fixed value. These features hinder the accurate prediction of ECG signals. Several papers have discussed the prediction of ECG signals. Wei et al. [15] developed a model for highly accurate prediction of ECGs and EEGs by combining CNN and bidirectional recurrent neural network (BRNN). In [16], Sun et al. proposed a prediction method of an ECG signal using an error backpropagation neural network (BPNN) and VMD technology. Sun et al. [17] proposed another prediction method of an ECG signal. The method was based on PSR theory and BPNN, with accuracy close to that of the previously mentioned method. To reduce the amount of data transmission, a waveform prediction lightweight algorithm was proposed in [1]. To improve the prediction accuracy, the algorithm used wavelet transform (WT) to preprocess the noise. Liu [18] proposed a data fusion algorithm based on WT and a least-squares support vector machine (LS-SVM). The algorithm used the LS-SVM model to predict an ECG signal. In [19], Heurtefeux et al. investigated the tradeoff between accuracy and complexity to predict ECG values in BANs. They suggested using an ARMA model to predict ECG values, but they could not build an ARMA model to predict ECG signals experimentally. Sun et al. [20] used an ARIMA model to analyze ECG data streams, but they did not use one to predict an ECG signal. An ECG signal prediction method based on an ARIMA model and a discrete wavelet transform (DWT) was proposed in [21]. This method obtained a good prediction effect, but it needed a significant amount of calculation because it used many high-order autoregressive (AR) models. In [22], an ECG signal prediction method was proposed in which the PSR theory and TS fuzzy model were used to predict an ECG signal. The accuracy of the prediction is close to that of [16, 17].

To improve the prediction accuracy of ECG signals, we propose a novel hybrid prediction method for ECG signals using variational mode decomposition (VMD), PSR, and a RBF neural network. In this paper, we make the following primary contributions.

- We use VMD to decompose a nonstationary ECG signal into several relatively stationary intrinsic mode functions (IMFs), which is helpful to improve the prediction accuracy. The parameter *K* represents the number of IMF decomposed by VMD. We determine *K* by the ratio of the residual energy to original signal energy.
- We use PSR to optimize the input parameters of the RBF neural network. We determine the embedding dimension by false nearest neighbors (FNN) method. The embedding dimension is the input dimension of the RBF neural network. We determine the delay time by comparing the mutual information (MI) method with the experimental method.
- We propose a novel prediction method for ECG signals based on VMD, PSR, and a RBF neural network. Compared with other prediction methods, the proposed method improves the prediction accuracy of ECG signals.

The rest of this paper is organized as follows. In Section 2, we describe the theories of VMD, PSR, and the RBF neural network and propose a novel hybrid prediction method for ECG signals. The simulation experiments and the analysis of its results are presented in Section 3. In Section 4, we provide concluding remarks.

## 2. Methods

### 2.1. Variational Mode Decomposition

Variational mode decomposition (VMD) can decompose a nonstationary signal into several discrete band-limited IMFs around the center frequency, meeting the condition that the sum of the estimated bandwidths of each mode is the smallest [23]. The decomposition steps can be summarized as follows. The parameter *K* represents the number of modal components decomposed by VMD and it must be predetermined before VMD.

**Step 1 .**

Calculate the analytic signal of each modal function *u*_*k*_(*t*) by Hilbert transform

(1) $$\begin{array}{c} \left(\delta \left(t\right)+\frac{j}{\pi t}\right)*u_{k}\left(t\right). \end{array}$$

**Step 2 .**

Multiply the analytical signal by the estimated center frequency *e*^−*jw*_*k*_*t*^, and move it to the base frequency spectrum

(2) $$\begin{array}{c} \left[\left(\delta \left(t\right)+\frac{j}{\pi t}\right)*u_{k}\left(t\right)\right]e^{-{jw}_{k}t}. \end{array}$$

**Step 3 .**

Estimate the bandwidth of each mode by Gaussian smoothing of the demodulated signal, i.e., the *L*^2^ norm of the gradient. The constrained variational model is

(3) $$\begin{array}{c} \left\{\begin{array}{c} \min_{\left\{u_{k}\right\}\left\{w_{k}\right\}}\left\{\underset{k}{\sum }{\left\|\left[\left(\delta \left(t\right)+\frac{j}{\pi t}\right)*u_{k}\left(t\right)\right]e^{-{jw}_{k}t}\right\|}_{2}^{2}\right\}\\ \text{s}.\text{t}.\;\underset{k}{\sum }u_{k}=x, \end{array}\right. \end{array}$$

where *x* is the input signal and ‖·‖_2_ is the Euclidian distance.

**Step 4 .**

Turn the constrained variational model into an unconstrained variational model by introducing the quadratic penalty factor *α* and Lagrange multiplication operator *λ*(*t*). The extended Lagrange expression is

(4) $$\begin{array}{c} L\left(\left\{u_{k}\right\},\left\{w_{k}\right\},\lambda \left(t\right)\right)=\alpha \underset{k}{\sum }{\left\|\left[\left(\delta \left(t\right)+\frac{j}{\pi t}\right)*u_{k}\left(t\right)\right]e^{-{jw}_{k}t}\right\|}_{2}^{2}+{\left\|x-\underset{k}{\sum }u_{k}\right\|}_{2}^{2}+<\lambda \left(t\right),x-\underset{k}{\sum }u_{k}>. \end{array}$$

Find the saddle point of the extended Lagrange expression using the alternating direction multiplier method (optimization algorithm) to solve the minimization problem of (3). The saddle point is the optimal solution.

**Step 5 .**

Obtain the saddle point of the extended Lagrange expression by alternately updating *u*_*k*_^*n*+1^, *w*_*k*_^*n*+1^, and *λ*_*k*_^*n*+1^. The equations for this are

(5) $$\begin{array}{c} {\overset{⌣}{u}}_{k}^{n+1}\left(w\right)=\frac{\overset{⌣}{x}\left(w\right)-\sum_{i<k}{\overset{⌣}{u}}_{i}^{n+1}\left(w\right)-\sum_{i>k}{\overset{⌣}{u}}_{i}^{n}\left(w\right)+\overset{⌣}{\lambda }\left(w\right)/2}{1+2\alpha {\left(w-w_{k}^{n}\right)}^{2}}, \end{array}$$

(6) $$\begin{array}{c} w_{k}^{n+1}=\frac{\int_{0}^{\infty }w{\left|{\overset{⌣}{u}}_{k}^{n+1}\left(w\right)\right|}^{2}dw}{\int_{0}^{\infty }{\left|{\overset{⌣}{u}}_{k}^{n+1}\left(w\right)\right|}^{2}dw}, \end{array}$$

(7) $$\begin{array}{c} {\overset{⌣}{\lambda }}^{n+1}{\overset{⌣}{u}}_{k}^{n+1}\left(w\right)={\overset{⌣}{\lambda }}^{n}{\overset{⌣}{u}}_{k}^{n+1}\left(w\right)+\tau \left({\overset{⌣}{x}\overset{⌣}{u}}_{k}^{n+1}\left(w\right)-\underset{k}{\sum }\left|{\overset{⌣}{u}}_{k}^{n+1}{\overset{⌣}{u}}_{k}^{n+1}\left(w\right)\right|\right), \end{array}$$

where $\overset{⌣}{u}\left(w\right)$, $\overset{⌣}{\lambda }\left(w\right)$, and $\overset{⌣}{x}\left(w\right)$ are the Fourier transforms of the signals *u*(*t*), *λ*(*t*), and*x*(*t*), respectively.

**Step 6 .**

Repeat Step 5 until the convergence condition is reached

(8) $$\begin{array}{c} \underset{k}{\sum }{\left\|{\overset{⌣}{u}}_{k}^{n+1}-{\overset{⌣}{u}}_{k}^{n}\right\|}_{2}^{2}/{\left\|{\overset{⌣}{u}}_{k}^{n}\right\|}_{2}^{2}<\varepsilon . \end{array}$$

### 2.2. Phase Space Reconstruction

Dutch mathematician Floris Takens [24] proved that as long as the embedding dimension is large enough, the reconstructed attractor retains the topological properties of the original attractor. In other words, in the trajectory of the reconstructed space, the reconstructed phase space and differential homeomorphism of the original power system are maintained, and the phase space of the delay time reconstruction keeps the geometric structure of the original system, along with its dynamic characteristics.

For a time series *x*_1_, *x*_2_, ⋯, *x*_*N*_, if the delay time *τ* and embedding dimension *m* can be properly determined, then the phase space can be reconstructed as

(9) $$\begin{array}{c} X=\left[\begin{array}{c} X_{1}\\ X_{2}\\ \vdots \\ X_{M} \end{array}\right]=\left[\begin{array}{cccc} x_{1} & x_{1+\tau } & \cdots & x_{1+\left(m-1\right)\tau }\\ x_{2} & x_{2+\tau } & \cdots & x_{2+\left(m-1\right)\tau }\\ \cdots & \cdots & \cdots & \cdots \\ x_{M} & x_{M+\tau } & \cdots & x_{N} \end{array}\right], \end{array}$$

where *M* = *N* − (*m* − 1)*τ* is the length of the vector sequence and *X*_*i*_ (*i* = 1, 2, ⋯, *M*) is a point of the phase space.

When *X* is used as the input vector , the one-step prediction output vector *Y* shows as follows:

(10) $$\begin{array}{c} Y=\left[\begin{array}{c} Y_{1}\\ Y_{2}\\ \vdots \\ Y_{M} \end{array}\right]=\left[\begin{array}{c} x_{2+\left(m-1\right)\tau }\\ x_{3+\left(m-1\right)\tau }\\ \vdots \\ x_{N+1} \end{array}\right]. \end{array}$$

The key to PSR is to correctly determine the embedding dimension *m* and delay time *τ*. The delay time can be determined by the average displacement, autocorrelation function, complex correlation, and mutual information (MI) methods. Methods to determine the embedding dimension include false nearest neighbors (FNN) method, Cao method, and G-P algorithm. Common methods to determine the embedding dimension and delay time at the same time include the window embedding and C-C methods.

#### 2.2.1. Mutual Information Method

Given that there are two systems *S* = {*s*_1_, *s*_2_, ⋯, *s*_*n*1_} and *Q* = {*q*_1_, *q*_2_, ⋯, *q*_*n*2_}, the information entropy of *S* and *Q* is

(11) $$\begin{array}{c} H\left(S\right)=-\overset{n1}{\underset{i=1}{\sum }}P_{s}\left(s_{i}\right)\log_{2}P_{s}\left(s_{i}\right), \end{array}$$

(12) $$\begin{array}{c} H\left(Q\right)=-\overset{n2}{\underset{j=1}{\sum }}P_{q}\left(q_{j}\right)\log_{2}P_{q}\left(q_{j}\right), \end{array}$$

respectively, where *P*_*s*_(*s*_*i*_) and *P*_*q*_(*q*_*j*_) are probabilities of *s*_*i*_ and *q*_*j*_, respectively.

The joint entropy of *S* and *Q* is

(13) $$\begin{array}{c} H\left(S,Q\right)=-\overset{n1}{\underset{i=1}{\sum }}\overset{n2}{\underset{j=1}{\sum }}P_{sq}\left(s_{i},q_{j}\right)\log_{2}P_{sq}\left(s_{i},q_{j}\right), \end{array}$$

where *P*_*sq*_(*s*_*i*_, *q*_*j*_) is the joint probability of *s*_*i*_ and *q*_*j*_.

Given that *S* has been measured, the conditional entropy of *Q* is

(14) $$\begin{array}{c} H\left(\frac{Q}{S}\right)=\underset{i}{\sum }P_{s}\left(s_{i}\right)H\left(\frac{Q}{s_{i}}\right)=-\underset{i,j}{\sum }P_{s}\left(s_{i}\right)P\left(\frac{q_{j}}{s_{i}}\right)\log_{2}P\left(\frac{q_{j}}{s_{i}}\right)=-\underset{i,j}{\sum }P_{sq}\left(s_{i},q_{j}\right)\log_{2}P\left(\frac{q_{j}}{s_{i}}\right)=-\underset{i,j}{\sum }P_{sq}\left(s_{i},q_{j}\right)\log_{2}\frac{P_{sq}\left(s_{i},q_{j}\right)}{P_{s}\left(s_{i}\right)}=-\underset{i,j}{\sum }P_{sq}\left(s_{i},q_{j}\right)\log_{2}P_{sq}\left(s_{i},q_{j}\right)-\left(-\underset{i,j}{\sum }P_{sq}\left(s_{i},q_{j}\right)\log_{2}P_{s}\left(s_{i}\right)\right)=-\underset{i,j}{\sum }P_{sq}\left(s_{i},q_{j}\right)\log_{2}P_{sq}\left(s_{i},q_{j}\right)-\left(-\underset{i,j}{\sum }P_{s}\left(s_{i}\right)P\left(\frac{q_{j}}{s_{i}}\right)\log_{2}P_{s}\left(s_{i}\right)\right)=-\underset{i,j}{\sum }P_{sq}\left(s_{i},q_{j}\right)\log_{2}P_{sq}\left(s_{i},q_{j}\right)-\left(-\underset{i}{\sum }P_{s}\left(s_{i}\right)\log_{2}P_{s}\left(s_{i}\right)\underset{j}{\sum }P\left(\frac{q_{j}}{s_{i}}\right)\right)=H\left(S,Q\right)-H\left(S\right). \end{array}$$

The MI of *Q* and *S* is

(15) $$\begin{array}{c} I\left(Q,S\right)=\underset{j}{\sum }\underset{i}{\sum }P_{sq}\left(q_{i},s_{j}\right)I\left(q_{i},s_{j}\right)=\underset{j}{\sum }\underset{i}{\sum }P_{sq}\left(q_{i},s_{j}\right)\left(I\left(q_{i}\right)-I\left(\frac{s_{j}}{q_{i}}\right)\right)=\underset{j}{\sum }\underset{i}{\sum }P_{sq}\left(q_{i},s_{j}\right)\left(\log_{2}\frac{1}{P_{q}\left(q_{i}\right)}-\log_{2}\frac{1}{P\left(q_{i}/s_{j}\right)}\right)=\underset{j}{\sum }\underset{i}{\sum }P_{sq}\left(q_{i},s_{j}\right)\log_{2}\frac{P\left(q_{i}/s_{j}\right)}{P_{q}\left(q_{i}\right)}=\underset{j}{\sum }\underset{i}{\sum }P_{sq}\left(q_{i},s_{j}\right)\log_{2}P\left(\frac{q_{i}}{s_{j}}\right)-\underset{j}{\sum }\underset{i}{\sum }P_{sq}\left(q_{i},s_{j}\right)\log_{2}P_{q}\left(q_{i}\right)=-H\left(\frac{Q}{S}\right)-\underset{j}{\sum }\underset{i}{\sum }P_{q}\left(q_{i}\right)P\left(\frac{s_{j}}{q_{i}}\right)\log_{2}P_{q}\left(q_{i}\right)=-H\left(\frac{Q}{S}\right)-\underset{i}{\sum }P_{q}\left(q_{i}\right)\log_{2}P_{q}\left(q_{i}\right)\underset{j}{\sum }P\left(\frac{s_{j}}{q_{i}}\right)=-H\left(\frac{Q}{S}\right)+H\left(Q\right)=H\left(Q\right)-H\left(\frac{Q}{S}\right). \end{array}$$

According to (14) and (15), the following results are obtained.

(16) $$\begin{array}{c} I\left(Q,S\right)=H\left(Q\right)-H\left(\frac{Q}{S}\right)=H\left(Q\right)-\left(H\left(S,Q\right)-H\left(S\right)\right)=H\left(Q\right)+H\left(S\right)-H\left(S,Q\right)=I\left(S,Q\right). \end{array}$$

Given that *Q* is the delayed sequence of *S*, that is, *Q* = {*s*_1+*τ*_, *s*_2+*τ*_, ⋯, *s*_n1+*τ*_}, the MI of *S* and *Q* is

(17) $$\begin{array}{c} I\left(\tau \right)=I\left(S,Q\right)=I\left(Q,S\right). \end{array}$$

The *τ* corresponding to the first local minimum of *I*(*τ*) is the optimal delay time *τ*.

#### 2.2.2. False Nearest Neighbors Method

False nearest neighbors (FNN) are phase points that are adjacent in low-dimensional space, but not adjacent after mapping to a certain high-dimensional space. Suppose *X*_*m*_(*i*) = (*x*_*i*_, *x*_*i*+*τ*_, ⋯, *x*_*i*+(*m* − 1)*τ*_) is a phase vector in *m*-dimensional reconstruction space, and the nearest neighbor point of *X*_*m*_(*i*) is *X*_*m*_^*NN*^(*i*). If *X*_*m*_^*NN*^(*i*) satisfies the following inequality when the dimension of reconstruction space increases from *m* dimensions to *m* + 1 dimensions, *X*_*m*_^*NN*^(*i*) is the FNN of *X*_*m*_(*i*).

(18) $$\begin{array}{c} \frac{{\left\|X_{m+1}\left(i\right)-X_{m+1}^{NN}\left(i\right)\right\|}_{2}-{\left\|X_{m}\left(i\right)-X_{m}^{NN}\left(i\right)\right\|}_{2}}{{\left\|X_{m}\left(i\right)-X_{m}^{NN}\left(i\right)\right\|}_{2}}\geq R_{T}, \end{array}$$

where ‖·‖_2_ is the Euclidian distance and *R*_*T*_ is the threshold value.

As the embedding dimension *m* increases from small to large, the proportion of FNN points is calculated. When the proportion of FNN points is very small or the number of FNNs no longer changes as *m* increases, then *m* is the best embedding dimension.

### 2.3. RBF Neural Network

Unlike a BP neural network, which is a global approximation network, a RBF neural network is a kind of local approximation network. As long as there are enough hidden neurons, a RBF neural network can approximate any continuous nonlinear function with any accuracy. Compared with a BP neural network, a RBF neural network has the advantage of fast training speed, and it does not easily fall into local minima.

The basic idea of a RBF neural network is that the RBF of the hidden layer node transforms the input vector, maps the low-dimensional input data to the high-dimensional space, weights the sums of the output of the node, and maps the results from the high-dimensional space to the low-dimensional space for output [25].

**Figure 1:**
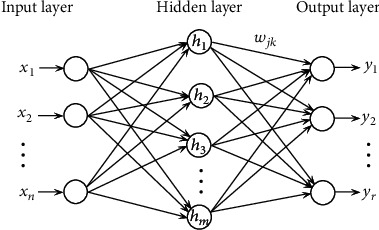
Structure of RBF neural network.

The network structure of a RBF neural network generally consists of an input layer, a hidden layer, and an output layer, as shown in Figure 1.

In Figure 1, a Gaussian function is usually used as the RBF of a hidden layer as follows.

(19) $$\begin{array}{c} h_{j}=\exp\left[-\frac{{\left(X-C_{j}\right)}^{2}}{2\sigma_{j}^{2}}\right],j=1,2,\cdots ,m, \end{array}$$

where *X* = [*x*_1_, *x*_2_, ⋯, *x*_*n*_], *C*_*j*_ is the center of the Gaussian function, *σ*_*j*_ is the variance of the Gaussian function, and *m* is the number of hidden layer nodes.

The output of the RBF neural network is

(20) $$\begin{array}{c} y_{k}=\overset{m}{\underset{j=1}{\sum }}w_{jk}h_{j}=\overset{m}{\underset{j=1}{\sum }}w_{jk}\exp\left[-\frac{{\left(X-C_{j}\right)}^{2}}{2\sigma_{j}^{2}}\right],k=1,2,\cdots ,r, \end{array}$$

where *w*_*jk*_ is the connection weight from the hidden layer to the output layer.

The learning algorithm of the RBF neural network solves for three parameters, which are the center of the RBF, the variance of the RBF, and the weight of the connection between the hidden layer node and output layer node. Common learning algorithms of RBF neural networks include *k*-means, the gradient training method, and the orthogonal least square product (OLS) algorithm.

### 2.4. Proposed ECG Signal Prediction Method

**Figure 2:**
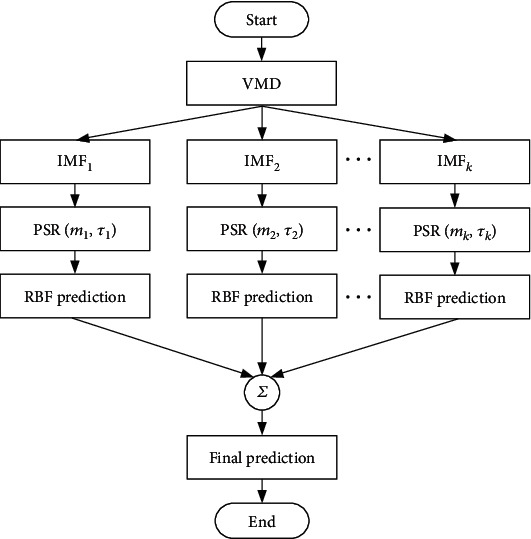
Flowchart of the proposed method.

Based on the study of ECG prediction, this paper proposes a hybrid method of ECG signal prediction based on VMD, PSR, and a RBF neural network. Its flowchart is shown in Figure 2.

The prediction steps of the proposed method are as follows.

Step 1. Decompose an ECG signal into *K* IMFs by VMD. In the experiment, we take *K* = 10 according to the ratio of residual energy to the original signal energy.

Step 2. Determine the embedding dimension *m* and delay time *τ* of each IMF. We determine the embedding dimension by the FNN method and determine the delay time by comparing the MI method with the experimental method.

Step 3. Reconstruct the phase space of each IMF according to the embedding dimension and delay time.

Step 4. According to the trained set of each IMF, establish a RBF neural network and use it to predict the test set of each IMF. The embedding dimension is the input dimension of the RBF neural network.

Step 5. Add the prediction results of the RBF neural network to obtain the final ECG signal prediction result.

Step 6. Analyze the prediction error and compare it with some traditional prediction methods.

## 3. Results and Discussion

All ECG data in the simulation experiment are from the MIT-BIH Arrhythmia Database [26]. We randomly selected No. 100 ECG data, which consists of 2768 data points, for the experiment. We used two-thirds of the source data as the trained set (1845 data) and one-third of the source data as the test set (923 data).

### 3.1. Performance Measures

The common performance measures of prediction methods are root mean square error (RMSE), mean absolute error (MAE), and mean square error (MSE), defined as

(21) $$\begin{array}{c} \text{RMSE}=\sqrt{\frac{1}{N}\overset{N}{\underset{n=1}{\sum }}{\left(X\left(n\right)-\overset{⌣}{X}\left(n\right)\right)}^{2}}, \end{array}$$

(22) $$\begin{array}{c} \text{MAE}=\frac{1}{N}\overset{N}{\underset{n=1}{\sum }}\left|X\left(n\right)-\overset{⌣}{X}\left(n\right)\right|, \end{array}$$

(23) $$\begin{array}{c} \text{MSE}=\frac{1}{N}\overset{N}{\underset{n=1}{\sum }}{\left|X\left(n\right)-\overset{⌣}{X}\left(n\right)\right|}^{2}, \end{array}$$

where $\overset{⌣}{X}\left(n\right)$ is the predicted value of *X*(*n*) and *N* is the length of *X*(*n*).

### 3.2. VMD of ECG Signal

A key problem of VMD is to set the number *K* of modal components. *K* affects the accuracy of the final prediction. If *K* is too small, the original series may not be decomposed sufficiently. If the signal is too large, the difference between each component becomes very small, and it gives rise to unnecessary computing overhead. *K* can be determined according to the ratio *R*_res_ of residual energy to the original signal energy. *R*_res_ is defined as follows:

(24) $$\begin{array}{c} R_{\text{res}}=\frac{1}{N}\overset{N}{\underset{n=1}{\sum }}\left|\frac{X\left(n\right)-\sum_{k=1}^{K}u_{k}\left(n\right)}{X\left(n\right)}\right|, \end{array}$$

where *X*(*n*) is the original signal, *u*_*k*_ (*n*) is the IMF, and *N* is the sample number. The decision rule of *K* is that when *R*_res_ is less than 1% and there is no significant downward trend, the number *K* can be determined [27]. For the No. 100 ECG data, *R*_res_ of VMD with different *K* are shown in Table 1.

Table 1 shows that *R*_res_ has no obvious downward trend when *K* = 10. Therefore, we set *K* = 10 in the experiment.

**Figure 3:**
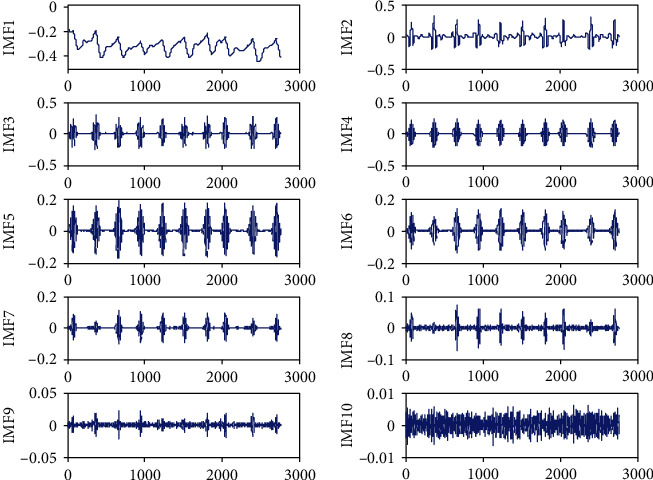
VMD of ECG signal.

The No. 100 ECG was decomposed into 10 IMFs by VMD as shown in Figure 3. IMF1 is the residual, and IMF2-IMF10 is the component, sorted from low to high frequency.

### 3.3. Determination of Delay Time and Embedding Dimension

**Figure 4:**
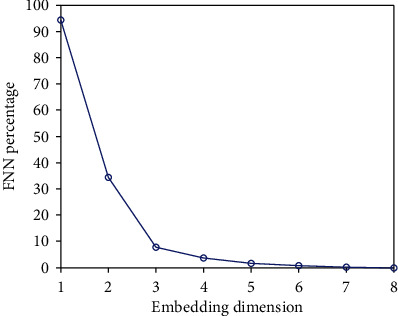
Determining embedding dimension by FNN method.

We determined the embedding dimension *m* and delay time *τ* of each IMF. The embedding dimension *m* of IMF3 was determined by the FNN method, as shown in Figure 4.

**Figure 5:**
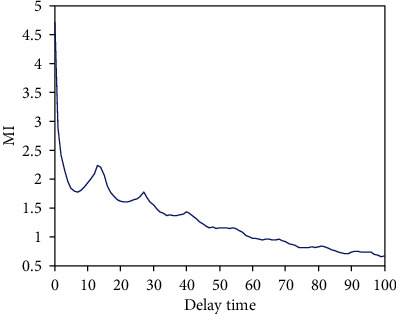
Determining delay time by the MI method.

From Figure 4, we obtained *m* = 5. The delay time *τ* of IMF3 was determined by the MI method, as shown in Figure 5.

In Figure 5, we obtained the delay time *τ* = 7 according to the first local minimum of MI. The embedding dimension and delay time of each IMF are shown in Table 2.

In the experiment, we obtained better prediction results when the delay time of each IMF was 1 (i.e., *τ* = 1). The comparison results are shown in Table 3.

According to the comparison in Table 3, we finally took the delay of each IMF as 1 (i.e., *τ* = 1).

### 3.4. Optimization of RBF Neural Network

In the simulation experiment, we used MATLAB function *newrbe(P*,*T,spread)* to design a RBF neural network. The parameter *P* is the input vectors of the RBF network, the parameter *T* is the output vectors of the RBF network, and the parameter *spread* is the spread of the RBF network. We used PSR to optimize *P* and *T*, that is, we used equations (9) and (10) to determine *P* and *T*, respectively. When the parameters *P* and *T* had been determined, we determined the parameter *spread* by experiments. Some experimental results are shown in Table 4.

We carried out the simulation experiment in the range of [0.1, 100] of the parameter *spread*. The best prediction results in the simulation experiment are RMSE = 0.0035 and MAE = 0.0027 when spread = 0.4, 0.5, and 0.6.

### 3.5. Prediction Results of the Test Set

**Figure 6:**
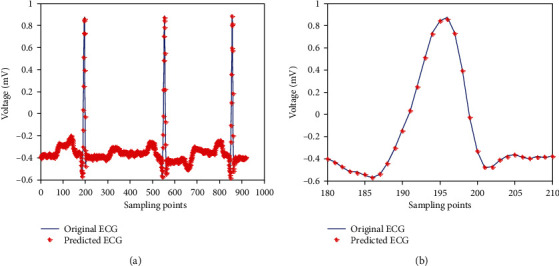
Prediction waveform: (a) prediction waveform of test set; (b) a part of prediction waveform of (a).

We used the proposed method to predict the test set of the No. 100 ECG signal. The prediction waveform is shown in Figure 6.

Figure 6(b) is a partial ECG signal amplification from [180 : 210] in Figure 6(a). Figure 6 shows that the predicted waveform fits well with its original ECG waveform.

**Figure 7:**
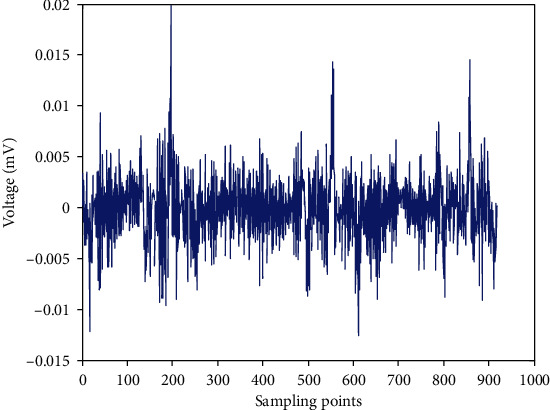
The prediction errors of test set.

The prediction errors are shown in Figure 7.

In Figure 7, in addition to the large error at the peak point, the error in other places is relatively small.

To evaluate the generality of the proposed method, we used ECG data other than the No.100 ECG data to carry out experiments. The experimental results are shown in Table 5.

**Figure 8:**
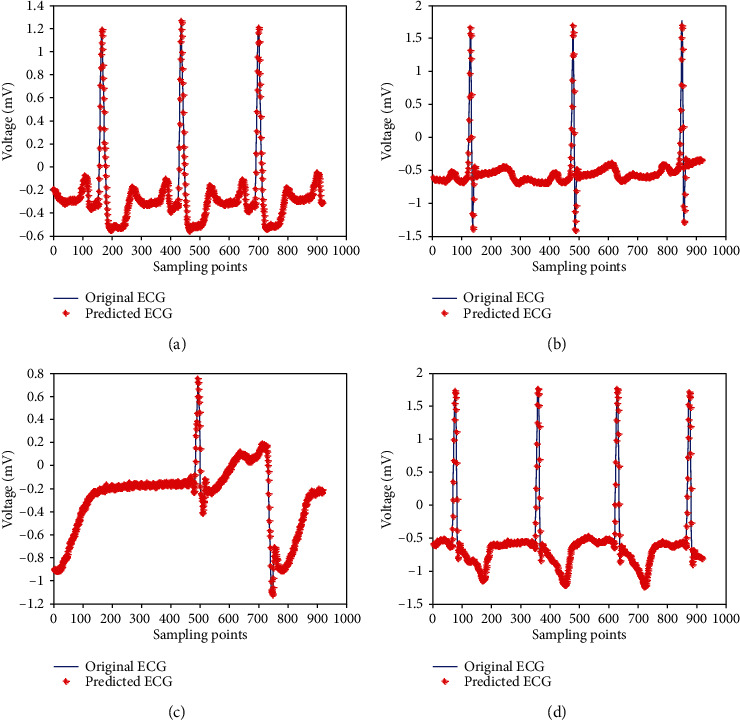
Prediction results of different ECG numbers: (a) No. 105; (b) No. 115; (c) No. 207; (d) No. 219.

Some prediction waveforms are shown in Figure 8.

From Table 5 and Figure 8, the prediction performances of the proposed method on other ECG data are very close to that of the No. 100 ECG data. This illustrates that the generality of the proposed method is good and it is very suitable for ECG signal prediction.

### 3.6. Comparison with Other Prediction Methods

**Figure 9:**
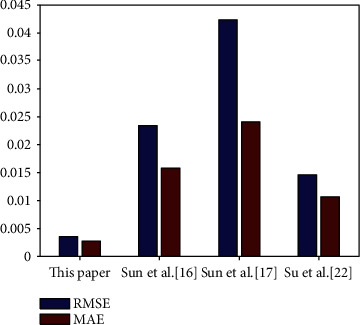
Comparison with common prediction methods.

To illustrate the advantages of the proposed prediction method, we compared it with the prediction methods of Sun et al. [16], Sun et al. [17], and Su et al. [22]. We experimented on the same data source, the No. 100 ECG data, and the experimental results are shown in Table 6 and Figure 9.

As shown in Table 6, the RMSE and MAE of the proposed method are of 10^−3^ magnitude, while the RMSE and MAE of [16, 17, 22] are of 10^−2^ magnitude. Table 6 and Figure 9 show that the RMSE and MAE of the proposed method are significantly smaller than those of [16], [17], and [22]. This illustrates that the prediction accuracy of this paper is much higher than that of [16, 17, 22].

We also compared the proposed method with some traditional prediction models, such as ARMA, LS-SVM, SVM, and Kalman. The experimental results on the data source of No. 100 ECG are shown in Table 7.

We can see from Table 7 that the RMSE of the proposed method is much smaller than that of LS-SVM, SVM, ARMA, and Kalman.

**Figure 10:**
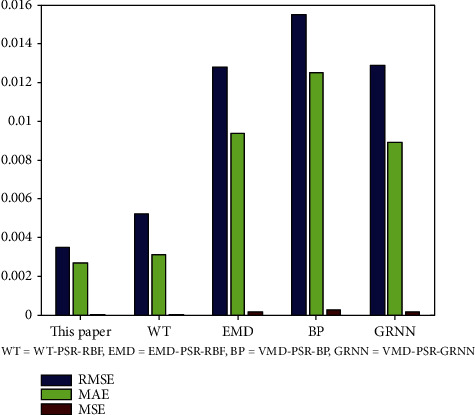
Comparison with some approximate hybrid methods.

In addition, the proposed method (VMD-PSR-RBF for short) was compared with some approximate hybrid prediction methods, such as the method based on WT, PSR, and a RBF neural network (WT-PSR-RBF for short), the method based on EMD, PSR, and a RBF neural network (EMD-PSR-RBF for short), the method based on VMD, PSR, and a BP neural network (VMD-PSR-BP for short), and the method based on VMD, PSR, and a GRNN (VMD-PSR-GRNN for short). The experimental data are the No. 100 ECG data, and the experimental results are shown in Table 8 and Figure 10.

Table 8 and Figure 10 show that the prediction accuracy is better than that of several other approximate hybrid prediction methods.

**Figure 11:**
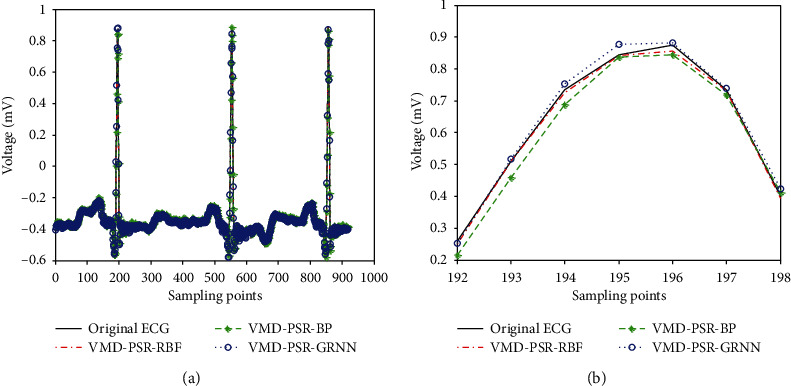
Comparison with VMD-PSR-BP and VMD-PSR-GRNN: (a) prediction waveform of test set; (b) a part of prediction waveform of (a).

**Figure 12:**
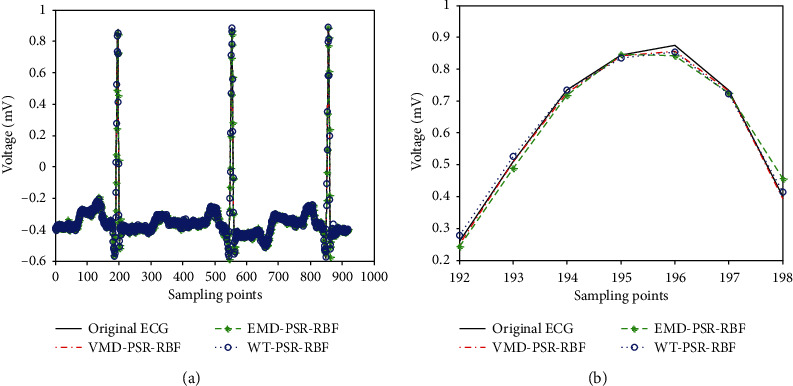
Comparison with EMD-PSR-RBF and WT-PSR-RBF.

The comparison prediction waveforms are shown in Figures 11 and 12.

Figure 11(b) is a partial ECG signal amplification from [192 : 198] in Figure 11(a). Figure 11(b) shows that the prediction curve of the proposed method (VMD-PSR-RBF) is closer to the original ECG curve than that of VMD-PSR-BP and VMD-PSR-GRNN.

As shown in Figure 12(b), the prediction curve of the proposed method (VMD-PSR-RBF) is also closer to the original ECG curve than that of EMD-PSR-BP and WT-PSR-RBF.

## 4. Conclusions

In this paper, we propose a hybrid prediction method for ECG signals using VMD, PSR, and a RBF neural network. Before prediction, we use VMD technology to preprocess the ECG signals in order to remove their nonstationarity and randomness. We determine the parameter *K* of VMD by the ratio of residual energy to original signal energy. To obtain the optimal input parameters of the RBF neural network, we employ PSR to determine the input dimension and the delay time of the RBF neural network. Using the ECG data from the MIT-BIH Arrhythmia Database as the data source, we evaluate the prediction performance of the proposed method. The RMSE and MAE of the proposed method are of 10^−3^ magnitude, while the RMSE and MAE of several common prediction methods are of 10^−2^ magnitude. The former is one order of magnitude smaller than the latter. The experimental results indicate that the proposed method is not only suitable for normal ECG signal prediction, such as No. 100 ECG, but also for abnormal ECG signal prediction, such as No. 207 ECG. Compared with other prediction methods, the proposed method is not only superior to some competitive prediction methods, such as [16, 17, 22], but also superior to some single prediction models, such as LS-SVM, SVM, ARMA, and Kalman. The proposed prediction method also outperforms some hybrid prediction methods, such as EMD-PSR-RBF and VMD-PSR-BP. WT-PSR-RBF is a competitive prediction method, its RMSE and MAE are 0.0052 and 0.0031, respectively, but the RMSE and MAE in this paper are smaller, only 0.0035 and 0.0027. As future work, we consider employing the prediction method proposed in this paper to reduce the energy consumption of the sensor in BANs.

## Figures and Tables

**Table 1:** *R*_res_ of VMD with different *K*.

*K*	1	2	3	4	5	6	7	8	9	10	11
*R*_res_	0.0658	0.0258	0.0116	0.0064	0.0035	0.0021	0.0018	0.0015	0.0018	0.0017	0.0017

**Table 2:** The parameters of each IMF.

Parameters	IMF1	IMF2	IMF3	IMF4	IMF5	IMF6	IMF7	IMF8	IMF9	IMF10
*τ*	31	18	7	5	4	3	2	2	1	3
*m*	4	4	5	5	5	5	5	4	4	4

**Table 3:** The comparison results of different delay times.

Delay time	IMF1	IMF2	IMF3	IMF4	IMF5	IMF6	IMF7	IMF8	IMF9	IMF10	RMSE	MAE
*τ*	31	18	7	5	4	3	2	2	1	3	0.0054	0.0041
*τ* ^∗^	1	1	1	1	1	1	1	1	1	1	0.0035	0.0027

**Table 4:** Some experimental results of the parameter *spread*.

*Spread*	0.1	0.2	0.3	0.4	0.5	0.6	0.7	0.8	0.9	1
RMSE	0.1115	0.0055	0.0037	0.0035	0.0035	0.0035	0.0036	0.0036	0.0036	0.0036
MAE	0.0346	0.0035	0.0028	0.0027	0.0027	0.0027	0.0027	0.0028	0.0028	0.0028

**Table 5:** Prediction performance of different ECG numbers.

ECG numbers	RMSE	MSE	MAE
100	0.0035	1.2323*e* − 05	0.0027
101	0.0064	4.1198*e* − 05	0.0033
103	0.0048	2.3258*e* − 05	0.0036
105	0.0026	6.8964*e* − 06	0.0020
115	0.0090	8.0978*e* − 05	0.0039
118	0.0082	6.6581*e* − 05	0.0037
123	0.0041	1.6513*e* − 05	0.0030
201	0.0025	6.0916*e* − 06	0.0018
207	0.0021	4.2296*e* − 06	0.0015
212	0.0043	1.8733*e* − 05	0.0033
219	0.0048	2.2814*e* − 05	0.0037
223	0.0036	1.3294*e* − 05	0.0028
234	0.0038	1.4448*e* − 05	0.0030

**Table 6:** Comparison with common prediction methods.

Methods	RMSE	MAE
This paper	0.0035	0.0027
Sun et al. [16]	0.0233	0.0157
Sun et al. [17]	0.0423	0.0240
Su et al. [22]	0.0146	0.0106

**Table 7:** Comparison with traditional prediction models.

Prediction models	RMSE
This paper	0.0035
LS-SVM [1]	0.1754
SVM [18]	0.0669
ARMA [18]	0.4630
Kalman [18]	0.3967

**Table 8:** Comparison with other hybrid prediction methods.

Prediction methods	RMSE	MSE	MAE
VMD-PSR-RBF (this paper)	0.0035	1.2323*e* − 05	0.0027
WT-PSR-RBF	0.0052	2.6576*e* − 05	0.0031
EMD-PSR-RBF	0.0128	1.6496*e* − 04	0.0094
VMD-PSR-BP	0.0155	2.3991*e* − 04	0.0125
VMD-PSR-GRNN	0.0129	1.6752*e* − 04	0.0089

## Data Availability

In this paper, all ECG data are from MIT-BIH Arrhythmia Database. MIT-BIH Arrhythmia Database is available online: https://www.physionet.org/content/mitdb/1.0.0/.
